# The role of mouse tumour models in the discovery and development of anticancer drugs

**DOI:** 10.1038/s41416-019-0495-5

**Published:** 2019-06-24

**Authors:** Christopher R. Ireson, Mo S. Alavijeh, Alan M. Palmer, Emily R. Fowler, Hazel J. Jones

**Affiliations:** 1 Pharmidex Pharmaceutical Services, 14 Hanover Street, London, W1S 1YH UK; 2 Reading School of Pharmacy, Whiteknights, Reading, RG6 6A UK; 3 Wellcome Centre for Cell Biology and Institute of Cell Biology, School of Biological Sciences, The University of Edinburgh, Edinburgh, EH9 3BF Scotland UK; 4 AstraZeneca, Oncology R&D, Cambridge, UK

**Keywords:** Cancer models, Metastasis

## Abstract

Our understanding of cancer biology has increased substantially over the past 30 years. Despite this, and an increasing pharmaceutical company expenditure on research and development, the approval of novel oncology drugs during the past decade continues to be modest. In addition, the attrition of agents during clinical development remains high. This attrition can be attributed, at least in part, to the clinical development being underpinned by the demonstration of predictable efficacy in experimental models of human tumours. This review will focus on the range of models available for the discovery and development of anticancer drugs, from traditional subcutaneous injection of tumour cell lines to mice genetically engineered to spontaneously give rise to tumours. It will consider the best time to use the models, along with practical applications and shortcomings. Finally, and most importantly, it will describe how these models reflect the underlying cancer biology and how well they predict efficacy in the clinic. Developing a line of sight to the clinic early in a drug discovery project provides clear benefit, as it helps to guide the selection of appropriate preclinical models and facilitates the investigation of relevant biomarkers.

## Background

Fundamental to the discovery and development of anticancer drugs is the ability to model tumour growth, recapitulating elements and characteristics of the human disease in mammalian organisms, and to demonstrate measurable effects of an anticancer drug. A drug can be defined, in its broadest sense, as a substance intended for use in the diagnosis, cure, mitigation, treatment or prevention of disease. Drugs for the treatment of cancer range  from cytotoxic agents, e.g. cisplatin, to the biological therapeutic Keytruda (pembrolizumab). Rodents, primarily mice, have been extensively used to increase our understanding of the aetiology and pathophysiology of human cancers, including phenotypic characteristics or hallmarks,^1^ as well as to facilitate the pharmacological evaluation of existing and potential new medicines.^2^

The success of models critically depends on the extent to which the positive efficacy data attained preclinically are predictive of efficacy in the clinic. In a review of oncology trials carried out in 2014, it was shown that a lack of validated preclinical models or an unclear disease linkage, defined in the simplest sense as a lack of association between the specific target and the disease state, was the most common reason that a drug failed to demonstrate clinical efficacy.^3^ A significant reduction on return of investment in the pharmaceutical industry was measured between 2010 and 2015,^4^ supporting the need to improve attrition rates, which are much higher for cancer than for other therapeutic areas. Indeed, attrition is substantial in the development of cancer therapeutics, with 95% of agents that enter phase 1 of clinical development, failing to gain market authorisation.^5^ These figures can be partly attributed to the poor predictive value of conventional preclinical models. Inadequate models will also inevitably lead to increased development times for new anticancer medicines, which, in turn, delays the provision of effective therapies to cancer patients, further underpinning the need to improve upon the *status quo*.

Careful consideration needs to be given to the validation and characterisation criteria of the models used. This will increase confidence in the models' applicability to disease and the potential for drug candidates to be translated into an effective medicine. Work in the field of animal models of psychiatric disorders has led to the classification and definition of criteria of model validation, and these principles can be applied to oncology. The general validity of a model has three components: first, face validity (phenomenological similarity to the modelled condition); second, construct validity (the model has a sound theoretical rationale); and third, predictive validity (prediction of efficacy in the clinic).^6^ Clearly, the best proof of a model’s value is its predictive validity.

Preclinical models have value across the discovery and development pipeline, initially building confidence in target biology (e.g. proof of mechanism), then understanding functional modulation and impact on tumour growth before defining the line of sight to the clinic. Simple models, such as subcutaneous models (xenografts or syngeneic), are crucial in the discovery phase, ensuring that the molecules have the appropriate pharmacology and activity in a biological system. Once drug candidates have been selected, more complex preclinical models become essential, providing an efficacy signal of sufficient magnitude to permit progression into clinical evaluation. Typically, evidence for efficacy comes from a range of models, rather than from a single model, and is defined by the biology of the target and the primary indication.^7^

Another key element in drug development in oncology is the increasing focus on patient selection. Preclinical models help to identify and validate associated biomarkers that can be used to select and/or stratify patients or as  pharmacodynamic (PD) biomarkers  to measure the biological effect of drug candidates. It is important to identify biomarkers as early as possible in the drug discovery programme; for example, a number of genetic strategies have been used to address this challenge, including single-nucleotide polymorphism sequencing and next-generation sequencing.^8^ The identification and validation of biomarkers extend through preclinical development, and when used to determine the efficacy of drug candidates (usually defined as tumour growth inhibition or regression), build sufficient evidence to enable clinical development.

Extensive research by both the academic and pharmaceutical communities over the past 30 years has greatly increased the range of mouse tumour models available. This review will focus on (i) the range of models available during the discovery and development of anticancer drugs, (ii) how these models add value to the drug discovery process and (iii) how, when selected and used appropriately to address the specific study aims, they can reduce the risk of failure in a tumour medicine research programme. It  will cover both the use of simple and complex preclinical models, with a specific focus on their application to metastasis and tumour heterogeneity.

## Identifying and selecting preclinical models

Firstly, the clinical setting, albeit constantly evolving during the course of the drug discovery project, should be clearly identified, including the patient population to be treated within a disease indication. Selecting the appropriate preclinical model that reflects this identified patient population, combined with good experimental design, will ensure that the results provide evidence to progress towards clinical development. Each of these models have advantages and disadvantages, as described in Table 1.

**Table 1:** List of preclinical models with description and value they can add to drug discovery

	Model	Description of the model	Advantages	Disadvantages	References
SIMPLE	Subcutaneous (heterotopic implantation)	Cancer cell lines are inoculated subcutaneously (s.c.) into the flank of a mouse. These can be syngeneic or xenograft	• Ease of engraftment and monitoring—can measure tumour growth with callipers externally • Time and cost effective experimental design	• Poor resemblance of tumour microenvironment • Limited metastasis to remote tissues	Kelland et al.^15^
	Orthotopic implantation (xenograft and syngeneic)	Xenografts: immortalised human cancer cell lines, initially derived from cancer patients and implanted in immunocompromised mice Syngeneic: mouse cancer cell lines inoculated into the equivalent strain of immunocompetent mice	• Good reproducibility for target validation and candidate selection • Orthotopic syngeneic models are representative of the clinical tumour’s microenvironment	• In vitro passaging of cells can lead to unrepresentative tumour histology and heterogeneity • Poor predictive relevance in later clinical development	Guerin et al. 2014^55^
COMPLEX	Patient-derived xenograft (PDX)	Freshly derived human tumours inoculated into immunocompromised mice	• Can be used as a wider efficacy screen • Can be utilised as a preclinical clinical trial (potentially demonstrates response in a heterogeneous population) • PDX models have value in identifying and validating a biomarker hypothesis • Clinical histology more closely recapitulated	• Lack of tumour microenvironment prevents testing of immunomodulatory agents • Subcutaneous, not orthotopic • Low engraftment rates for some types of tumours	Tentler et al.^19^; Bertotti et al.^22^
	Circulating tumour cell- derived PDX (CDX)	Development of tumours in immunocompromised mice from blood-circulating tumour cells	• Similar to PDX, with the advantage of developing models for indications difficult to biopsy or receive surgical samples (such as early-stage disease)	• Cells are rare and difficult to collect, persist for a very short time in circulation	Girotti et al.^56^; Kitz et al.^57^
	Humanised PDX	Freshly derived human tumours inoculated into mice that have a humanised immune system	• Similar to PDX, primarily used for immunotherapy efficacy studies	• Depletion of the original haematopoietic system • Six to twelve months immune deficiency • Graft vs. host disease	Li et al.^58^
	Personalised PDX (or avatar)	PDX models of tumour development for a specific patient, to investigate potential treatment options	• Similar to PDX, sometimes used for choice of secondary treatment for patients	• Has the same disadvantages as PDX with additional costs and time constraints	Pauli et al.^29^
	Organoid xenograft	3D culture of tumour and related cells to model tumour formation in vitro, can then be transplanted into the mouse orthotopically or subcutaneously	• Allows reproducible growth of tumours ex vivo. Amenable to high drug throughput screening • Allows more accurate modelling than simple models	• Potential adaption or cellular selection for the in vitro environment • Organoids must be transplanted to immunosuppressed mice	O' Rourke et al.^35^; Fumagalli et al.^59^
	Genetically engineered mouse models (GEMM)	Tumours spontaneously arise by the action of key drivers in immunocompetent mice	• Some elements of disease can be recapitulated over a longer time, involving multiple cell types and development in the organ of origin • Design of preclinical studies enables a range of efficacy endpoints from tumour volume to quantification of metastasis	• Development time 2–10 months, investment in colony breeding is needed • Same mouse strain, not much model complexity in terms of genetic variation • Synchronous overexpression or inactivation of oncogenes and TSGs results in reduced clonal heterogeneity • Metastasis difficult to study as animals were killed early due to heavy primary tumour load. Removal of primary tumour can circumvent this challenge	Day et al.^2^; Singh et al.^38^
	Somatic tumour models	Creation of cancer cell lines via genetic modification (using CRISPR, RNAi) of normal cells before transplantation into a host animal	• Shares many of the same advantages as GEMMs • May be faster to produce and allow greater mouse numbers to be studied • Can be extracted from or implanted into a range of mouse backgrounds to take advantage of genetic variation • Allows tracing of the implanted cells via simultaneous transduction with a reporter construct	• May not accurately model the early stages of disease due to use of strong cancer drivers • Requires either murine cancer cells or human cells into immunosuppressed mice	Oldrini et al.^43^; Rodriguez et al.^60^

**Fig. 1:**
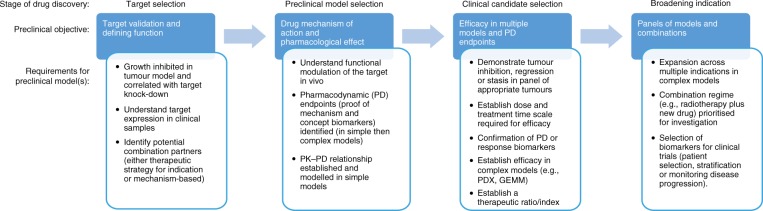
Schematic diagram showing the preclinical objectives and requirements for preclinical models to support preclinical development in key stages of drug discovery

Model selection, dosing and scheduling can be underpinned by earlier work, including analysis and evaluation of a broad panel of cell lines in vitro (e.g. the catalogue of somatic mutations in cancer (COSMIC) cell line project^9^), next-generation sequencing of tumour samples and analysis of clinical data sets. Models are firstly selected with a particular molecular subtype/proof-of-mechanism biomarker that is appropriate to the target, and how they reflect the pathology of the human disease. Target selection and preclinical validation, underpinned by an understanding of target expression in clinical tissue, can be facilitated by the use of simple subcutaneous or orthotopic models (Fig. 1). These models can either be xenografts or syngeneic, using immunocompromised or immunocompetent mice respectively.

Studies in complex models should be undertaken, once efficacy and an understanding of the mechanism has been demonstrated, and the target has been validated preclinically. The effect of the drug on the primary tumour can be determined using xenografts derived from patients’ solid tumours. Humanised patient-derived xenograft (PDX) models allow modelling of patient tumour heterogeneity in the presence of an intact human immune system, which is often particularly important in the development of immunotherapies.^10^ However, the majority of xenograft models have the disadvantage of using late-stage, malignant cancer cells, and therefore, cannot be used to model the early and stochastic development of tumours. Efforts are underway to address these factors by developing PDX models which represent early-stage disease, and by the use of genetically engineered mouse models (GEMMs) and organoid xenografts.

Confidence in selecting a drug candidate can be built using multiple tumour models. In a National Cancer Institute review, comparing the activity of 39 drug candidates with both xenograft data and the available phase 2 clinical trial results, in vivo activity, as shown by a particular histology in a tumour model, did not closely correlate with activity in the same human cancer histology. However, for compounds with in vivo activity in at least one-third of the tested xenograft models, there was evidence that the models had some predictive validity.^11^ This implies that whilst activity in multiple xenograft studies predicts clinical response, it is not necessarily true for particular disease indications. Currently, with many immunotherapies being developed and requiring panels of syngeneic models, it is essential to characterise the models fully. However, the translation of drug efficacy in these models into outcomes in clinical trials is, and should be, treated with caution, at least in part, due to the disadvantages described in Table 1.

In oncology clinical trials, overall survival, progression-free survival, time to progression and overall response rate are used as endpoints. In xenograft studies, by contrast, instead of endpoints defined by outcomes, measurable quantitative values include inhibition or delay of tumour growth.^11^ For example, the Hedgehog pathway inhibitor, vismodegib, inhibited the growth of tumours by > 60% in a preclinical model of medulloblastoma^12^ and subsequently elicited complete responses in paediatric metastatic medulloblastoma patients in a phase 1 study.^13^ By contrast, vismodegib inhibited the growth of tumours in a preclinical colorectal cancer xenograft model by 40%, but did not show any clinical benefit in a phase 2 clinical study. This exemplifies the urgent requirement for well-characterised endpoints in preclinical models, agreed by the scientific community, that ultimately should correlate with responses in the clinic.^14^

**Fig. 2:**
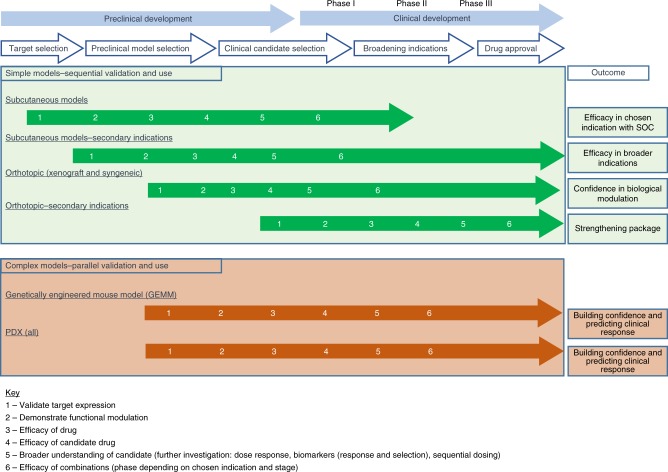
Selection of preclinical models to aid transition of drug discovery into clinical development

It is, of course, not feasible to simplify the selection of preclinical models into a plan that can be applied to all drug discovery projects. However, it is possible to depict the stage of the drug discovery pipeline at which each of the models can be used  (Fig. 2). Importantly, the overall outcome that can be achieved from studies in each of these models is shown and how these outcomes can be subdivided into incremental milestones (1–6), which are achieved at various stages of the drug discovery process. It is advisable, at the target selection and preclinical model selection of a project, to use simple models sequentially, until sufficient confidence is reached to progress into a larger panel of multiple indications or complex models. At later stages of preclinical development, complex models should be validated in parallel, until one shows utility to progress with as a workhorse. Although success criteria for each study will be defined by relevant controls and comparisons, decisions to progress a project are more defined by the level of confidence. For example, activity in one xenograft study is not sufficient, but if models are limited for a certain patient population, several simple models demonstrating efficacy and function alongside one complex model could support clinical development.

## Simple models

In this section, the use of simple models is discussed and their critical role in target selection and validation is considered (Fig. 1).

### Traditional subcutaneous cell-line xenograft and syngeneic models

The simplest tumour model is the subcutaneous model, in which cell lines initially derived from human tumours (xenograft model) or mouse tumours (syngeneic model) are typically inoculated subcutaneously into the flank of a mouse, and tumour growth is monitored with callipers. This approach represents a cost- and time-effective experimental design,^15^ and has been widely used during preclinical drug development since 1972. However, these models do not reflect the complexity of the multiple origins and functions of cells in the original tumour and its environment, particularly given their injection subcutaneously rather than into the corresponding initial anatomical position (heterotopic vs. orthotopic). Furthermore, as a consequence of their synchronous inoculation into mice, these models do not reflect the multistage process of carcinogenesis; this process can be recapitulated in complex models, predominantly GEMMs, although only certain elements of the clinical disease are captured. As each cell-line-derived xenograft/syngeneic model cannot represent the complexity of the human disease, it would not be expected that positive data attained from one tumour model can predict efficacy in a diverse patient population. The critical consequence of which would be the potential progression of molecules to clinical development, which, despite having robust preclinical data in a preclinical model, ultimately fails to demonstrate efficacy in the clinic.

Profiling of different syngeneic models demonstrates the importance of characterising a model before use for immunotherapy drug development,^16^ owing to differences in tumour immune cell infiltrates between models, and enables rational model selection. Furthermore, these authors showed that the response to immune checkpoint blockade was underpinned by cytotoxic effector immune cells. Whilst considering the caveats described, these models provide an opportunity to address the critical question of whether the mechanism of drug action aligns with the proposed therapeutic effect (Fig. 1), and to test pharmacological activity, pharmacokinetics (PK) and toxicity. In addition, these relatively cost-effective models enable the establishment of candidate PK–PD relationship, underpinning selection of the optimal dosing regimen in clinical trials and determination of the therapeutic ratio.^17^ However, as discussed, traditional subcutaneous xenografts have come under scrutiny over the past decade, because of both poor predictive validity and their limited relevance to the tumour type from which they are derived. The advantages and disadvantages of these models are described in Table 1.

## Complex models

Complex models, which are designed to recapitulate the clinical disease setting and may be more predictive of clinical efficacy, provide further confidence for decision-making in clinical development and clinical candidate selection (Fig. 1).

### Patient-derived xenograft (PDX) models

PDX models, in which freshly resected human tumours are implanted into immunosuppressed mice (e.g. nonobese diabetic (NOD)/severe combined immunodeficiency (SCID)), are one solution to circumvent some of the challenges associated with the use of cell-line xenografts. PDX models recapitulate more elements of tumour biology, tumour pathology, gene expression and mutations^18^ than subcutaneous cell line models, and thus have considerable utility in the selection of late preclinical/early clinical-stage patients that are most likely to respond to a new therapeutic agent.^19^ As these tumours are generally well characterised, both molecularly and histologically, it is possible to select those with a particular molecular subtype that aligns with the agent’s mechanism of action. Critically, this enables the particular project hypothesis to be tested in vivo, hence increasing confidence in the target and justifying the financial resource of commencing clinical development. Alternatively, a bank of PDX models can be used as a preclinical–clinical trial, representing a heterogeneous patient population and the corresponding activity.^20^

Panels of PDX models have been established by academic institutions, contract research organisations and pharmaceutical companies. Substantial efforts are being made to build PDX collaborative consortia, such as the European EuroPDX Consortium (http://europdx.eu/news-events.html) and collaborations with industry, which will increase the use of these models and potentially decrease study costs,^21^ as well as increasing the efficiency of animal usage within the 3Rs (Replacement, Reduction and Refinement) framework for the ethical use of animals in testing. This uses the breadth of models now available to evaluate efficacy across a heterogeneous population, and in some retrospective studies, the response in PDX models is predictive of clinical response, the most prominent amongst which are inhibitors of the epidermal growth factor receptor (EGFR), which prevent receptor activation of EGFR + tumours. Colorectal tumours with high EGFR copy number responded to cetuximab in both preclinical PDX models and clinical trials.^22^ The use of a nab-paclitaxel plus gemcitabine combination in pancreatic ductal adenocarcinoma^23^ also demonstrated success in a phase 3 clinical study, after synergistic antitumour activity was observed preclinically using PDX models.

However, there are still a number of limitations to PDX models. First, the cost of using and maintaining PDX panels is high. Second, as these tumours are human in origin and generally engrafted into immunodeficient mouse strains, these models cannot be used for testing immunomodulatory agents. Finally, most contract research organisations and academic groups have largely focused on subcutaneous tumours and have not investigated whether orthotopic tumours, in which tumours are grown in their organ of origin and hence their endogenous microenvironment, are more predictive of the clinical response than their heterotopic equivalent. This may be due to engraftment of tumours orthotopically being more technically challenging, with an associated reduction in engraftment rate.^24^

### Humanised PDX model

There is considerable interest in the use of PDX models for the development of immunotherapeutic agents, but the lack of a functional immune system in standard PDX models makes them unsuitable. A number of strategies have therefore been taken to establish a human immune system in immunodeficient rodents to generate humanised PDX models: implantation of peripheral blood mononuclear cells, containing T cells, B cells, natural killer cells and monocytes, alongside the tumours^25^; implantation of fresh tumour containing human stromal and immune cells^26^; implantation of tumours into mice into which human CD34+ haematopoietic stem cells have previously been transplanted following sublethal irradiation^27^ to replace the full haematopoietic system, including innate and adaptive immunity. This methodology has been used to characterise the response of a triple-negative breast PDX model to a combination of the PD-1 inhibitor nivolumab and a histone deacetylase inhibitor.^28^

### Personalised PDX models

In addition to their use in drug discovery, PDX models can be used for a personalised patient-centric approach to identify alternative therapeutic strategies for those patients who have not responded to standard-of-care treatment options^29^ (Table 1). Cells can be taken from the patient’s primary tumour site or circulating tumour cells (CTCs—which allows sampling of tumours without biopsy and can model metastatic pioneer cells) for xenotransplantation into immunosuppressed mice. This produces a model of the tumour that can be used to determine patient-specific drug response^30^ and potential identification of novel treatment strategies. However, not all tumours are amenable to xenotransplantation, the immune response is not modelled and the methodology may not facilitate transplantation of important treatment-resistant cells.^31^

### Organoid xenografts

Organoids can be created either via mechanical digestion of human tumour biopsies followed by in vitro growth, or via directed differentiation of ESCs/IPSCs,^32^ to form bodies that model the tumour microenvironment in 3D culture.^33^ Organoids offer a means for humanised high-throughput drug screening in vitro^34^ and have potential as reproducible human xenografts for use in drug treatment pipelines. In organoid studies of colorectal cancer (CRC), tumour progression mimics human CRC with histopathological accuracy and metastasis,^35^ and in one study shows a 96% mutational spectrum overlap with a parental biopsy. These early data show promise for overcoming some of the limitations associated with modelling the tumour microenvironment. These models may be used to predict drug response in patients, as exemplified by cetuximab in colorectal cancer.^36^

### Genetically engineered mouse models (GEMMs)

GEMMs, in which tumours spontaneously arise by the action of key human drivers in immunocompetent mice, might more closely recapitulate human disease than subcutaneous models or PDX models, as the stochastic and early development of tumours are features in this model.^37^ Studies involving GEMMs frequently use the overall survival of mice untreated and treated with the standard-of-care agent as translational endpoints. Proof-of-principle retrospective studies of standard of care in combination with an EGFR and vascular endothelial growth factor (VEGF) inhibitor have been completed in mutant KRAS-driven GEMMs of non-small-cell lung cancer (NSCLC) and pancreatic ductal adenocarcinoma (PDAC).^38^ These models successfully predicted clinical efficacy. Notably, the correlation between KRAS-driven mutations and response in NSCLC was not predicted using xenograft models,^39^ highlighting the importance of this strategy. The difference in therapeutic response between the GEMM and xenograft model could be attributed to a number of factors, including cell proliferation rates, relative tumour expression of KRAS and differences in dose scheduling, although this has not been experimentally confirmed.

Despite emerging data supporting the ability of GEMMs to better predict clinical response, their uptake in drug development by the pharmaceutical industry has been limited. This can be explained, at least in part, by the complex set-up required to manage these preclinical studies, as tumours can require 2–10 months to reach an appropriate size for therapeutic intervention, therefore requiring substantial investment in a breeding colony. Centralising models might be one solution to this challenge, in an analogous manner to the PDX consortia. Finally, the increasing prevalence and improvements to CRISPR technologies are allowing rapid and cost-effective creation of new GEMM models, which allow the study of complex genotypes.^40,41^ Alternatively, allografts, in which a fragment of a tumour is grafted into the same strain of mouse, represent a practical solution to decreasing the complexity and logistical challenges of GEMMs, as the experimental study length is reduced, due to direct tumour implantation, as opposed to waiting for spontaneous tumours to arise. Validation work is ongoing in a number of indications to confirm that the response of each allograft to standard-of-care treatment is equivalent to the original model from which the allograft was derived.

### Somatic tumour models

Somatic tumour models involve production of cancer cell lines from healthy murine or human tissue, typically via CRISPR-targeted genetic alteration of known oncogenes/tumour suppressors.^42,43^ These can then be transplanted into recipient mice. These models share many of the advantages of GEMMs, but with much reduced development time and are more likely to be predictive of clinical response, but this needs to be determined  experimentally.

## Investigating metastasis

Tumour metastasis to distal sites is associated with poor patient prognosis, and usually means a diagnosis of terminal illness. Metastasis is the leading cause of cancer death; ~90% of cancer patients who succumb to their illness die of metastatic disease, even though half of them initially present with only a localised tumour.^44^ The process of metastasis, however, is inefficient and most cells that break away from the initial primary tumour die; those that survive learn to adapt to restricted levels of oxygen, nutrients and space. Despite the urgent clinical need for drugs that target the multiple stages of the metastatic process,^45^ anti-metastatic drug development has been deprioritised by the pharmaceutical industry and academic community.

At a 2016 international workshop, it was concluded that there is a lack of relevant animal models that closely recapitulate clinical tumour dissemination and pathogenesis driven by clinically appropriate and quantitative endpoints.^46^ The models available are considered primarily to be tools for candidate selection rather than for predicting clinical efficacy, reflecting the current need to understand the complex biological processes involved in metastasis. The lack of preclinical models is attributable, at least in part, to the fact that subcutaneous tumour models rarely metastasise. Due to the many years during which a primary tumour may metastasise, it is likely that an effective therapy for metastasis will require chronic dosing and will be offered as part of a combination therapy. Demonstrating safety—particularly the absence of major organ toxicity—alongside efficacy within in vivo models is, therefore, important. A key factor in the design of in vivo studies investigating metastasis is determining the point at which to treat the tumours and how this reflects the clinical scenario, as tumour cells can be dormant for decades, maintained at a subclinical level by immunosurveillance, before giving rise to new tumours after adaptation at a secondary site.

Although syngeneic mouse models using 4T1 and E0771 mouse mammary tumour cells^47^ and colorectal tumours transplanted onto the colonic mucosa^48^ have been established and demonstrated to undergo spontaneous robust metastasis to the liver and bone, this is not the case for most tumour models. Tumours derived from breast cancer patients and inoculated directly into NOD/SCID mice, in a PDX model, have been shown to metastasise to the auxiliary lymph node and lung, faithfully recapitulating the metastatic profile of the human disease.^18^ Other studies have shown that MDA-MB-231 and MDA-MB-468 are also robust breast cancer models of metastasis. In most cases in these models, however, the tumour microenvironment is not recapitulated, which is critical for the multistage process of metastasis.^46^ By contrast, GEMMs facilitate the investigation of agents that intervene with the early stages of metastasis. Tumours in the KPC GEMM of pancreatic cancer^49^ have been shown to metastasise to the liver with a frequency of 50–75% and have been used to show that the src kinase inhibitor, dasatinib, inhibits the development of metastasis.^50^

In conclusion, although the clinical management and treatment of metastasis are critical to patient survival, the use of models in drug discovery to investigate the process of metastasis is limited. However, current models might provide key mechanistic and pharmacological information to drive a lead candidate forward to clinical development to address the significant unmet clinical need.

## Tumour heterogeneity and drug response

Historically, it has been difficult to study drug responses in the context of the tumour microenvironment, due to the complexity and heterogeneity of tumours. In addition to inter-tumour heterogeneity (tumours from different patients with divergent genetic backgrounds and cell surface markers), intra-tumour heterogeneity is a therapeutic challenge. Tumours consist of cancer stem cells, malignant and normal cells, stroma, immune cells and growth factors, which combine to form an array of both tumour-suppressive and oncogenic-interlinking systems. Differences in complex signalling cascades and interactions mean that tumours, even within a single patient, are genetically, phenotypically and clinically heterogeneous. Humanised PDX models used to test a drug in a diverse panel of patient-derived tumours in the context of an intact immune system will facilitate an understanding of the relationship between different mutational backgrounds and the drug response, for example in melanoma.^51^ These models, characterised by their genomic profile and protein expression, enable clustering of patient tumour into molecular subtypes, which correlate to different treatment regimens.

To understand the influence of intra-tumour heterogeneity upon clinical outcomes, longitudinal sampling strategies to molecularly characterise tumour subclones are being carried out. The TRAcking Cancer Evolution through therapy ((Rx); TRACERx) consortia is focusing on renal,^52^ NSCLC,^53^ melanoma and prostate cancer. In these studies, underpinned by next-generation sequencing and histology, tumour evolution with time, and ultimately clinical response to treatment, can be investigated using minimally invasive sampling methods. These data will facilitate the identification and development of novel preclinical models and therapeutic strategies.

## Conclusions

Preclinical models critically underpin all of the stages of the progression of a drug candidate through drug discovery and development towards regulatory approval and final marketing as a licenced medicine (Fig. 1). A clear efficacy signal in a tumour model is essential for target validation. It also increases the probability of attaining a robust efficacy response in the clinic. This objective can be achieved by initially showing a tumour response in simple subcutaneous models, before subsequently building confidence in efficacy studies in complex models and characterising the effects in selected indications or disease populations.

Selection of the optimum complex tumour models is governed by the mechanism of action of the drug candidate and the scientific justifications for the planned clinical route. As a full suite of models is not currently available, this can range from no models to a bank of PDX models or well-characterised GEMMs (Table 1). Building on justification of biomarkers and combinations should start early and continue through clinical development, and is now a more iterative process, cycling through preclinical and early clinical development, rather than sequential steps of drug development.^54^ Furthermore, window-of-opportunity studies are enabling mechanistic understanding earlier in the developmental process.

The models described and critically evaluated in this review enable three key aims to be achieved during the drug discovery and development process. Firstly, target engagement is confirmed and modulation correlated with tumour response before validating the target in the preclinical setting, typically in simple models, and identifying PD biomarkers. Secondly, complex models (e.g. PDX and GEMM) can be used to give confidence in different disease indications—efficacy in one model does not necessarily translate into efficacy in other models. The degree of confidence depends on characterisation of the model linking clinical aetiology with endpoints and supports the declaration of a clinical candidate and its clinical development. These complex models more closely recapitulate human tumour histopathology, and putatively, response to therapeutic agents, although this remains to be unequivocally experimentally verified. Thirdly and finally, during clinical development, preclinical models facilitate the rational selection of multiple indications, combinations of therapeutics and optimisation of PD biomarkers of response. Clearly, any target can only be fully validated on the basis of successfully predicting clinical efficacy. Nonetheless, selecting the right preclinical model is likely to reduce the risk of failure in clinical development, provided that the right scientific question is being asked.

In conclusion, over the last decade, the array of preclinical oncology models has expanded substantially. Such models enable confirmation of target engagement and mechanism of action of the drug candidate, along with the demonstration of in vivo efficacy. In conjunction with their associated biomarkers, they have the potential to greatly facilitate and accelerate the delivery of new medicines to people with cancer.

## Data Availability

Not applicable.
